# Results of a Modified Sugiura's Devascularisation in the Management of “Unshuntable” Portal Hypertension

**DOI:** 10.1155/1999/59087

**Published:** 1999-07

**Authors:** S. R. Shah, S. S. Nagral, S. K. Mathur

**Affiliations:** 1 Department of Surgery and Gastroenterology Surgical Services King Edward VII Memorial Hospital Bombay 400 012 India; 2 123, Khushnuma 29A Carmichael Road Bombay 400 026 India

## Abstract

The results of a modified Sugiura devascularisation procedure were assessed in 14 patients with thrombosis of the portal and splenic vein requiring surgery for variceal hemorrhage, with no vein suitable for orthodox shunt surgery. The venous anatomy was determined by ultrasonography with Doppler studies and portovenography. Liver biochemistry as well as liver architecture on histopathology was normal in all. The surgery was elective in 9 cases for documented bleed from diffuse fundal gastric varices (FGV) and emergency in 5 cases, 3 having bleeding FGV and 2 for failure of emergency esophageal variceal sclerotherapy. All were subjected to a transabdominal extensive devascularisation of the upper two third of the stomach and lower 7–10cm of the esophagus. Stapled esophageal transection (*n*=11) or esophageal variceal under-running (*n*=1) was performed in all with esophageal varices. FGV were underrun. Follow up endoscopies were done six monthly. There were 9 males and 5 females with a mean age of 17.2 years (SD 12.8). There was no operative mortality. Acute variceal bleeding was controlled in all patients. Over a mean follow up of 38 months, all but one remain free of recurrent bleeding. We conclude that a modified Sugiura devascularisation procedure is effective in the immediate and medium term control of variceal bleeding in patients with “unshuntable” portal hypertension.


*HPB Surgery*, 1999, Vol. 11, pp. 235-239
Reprints available directly from the publisher
Photocopying permitted by license only

© 1999 OPA (Overseas Publishers Association) N.V.
Published by license under
the Harwood Academic Publishers imprint,
part of The Gordon and Breach Publishing Group.
Printed in Malaysia.

### Results of a Modified Sugiura's Devascularisation in the Management of "Unshuntable" Portal Hypertension

S. R. SHAH*, S. S. NAGRAL and S. K. MATHUR

*Department of Surgery and Gastroenterology Surgical Services, King Edward VII Memorial Hospital, Bombay 400 012, India*

*(Received 30 November 1997; In final form 30 July 1998)*

**The results of a modified Sugiura devascularisation procedure were assessed in 14 patients with thrombosis of the portal and splenic vein requiring surgery for variceal hemorrhage, with no vein suitable for orthodox shunt surgery. The venous anatomy was determined by ultrasonography with Doppler studies and portovenography. Liver biochemistry as well as liver architecture on histopathology was normal in all. The surgery was elective in 9 cases for documented bleed from diffuse fundal gastric varices (FGV) and emergency in 5 cases, 3 having bleeding FGV and 2 for failure of emergency esophageal variceal sclerotherapy. All were subjected to a transabdominal extensive devascularisation of the upper two third of the stomach and lower 7-10 cm of the esophagus. Stapled esophageal transection ($n = 11$) or esophageal variceal underrunning ($n = 1$) was performed in all with esophageal varices. FGV were underrun. Follow up endoscopies were done six monthly. There were 9 males and 5 females with a mean age of 17.2 years (SD 12.8). There was no operative mortality. Acute variceal bleeding was controlled in all patients. Over a mean follow up of 38 months, all but one remain free of recurrent bleeding. We conclude that a modified Sugiura devascularisation procedure is effective in the immediate and medium term control of variceal bleeding in patients with "unshuntable" portal hypertension.**

*Keywords:* Portal hypertension, extrahepatic portal venous obstruction, devascularisation

Portal hypertension due to extra hepatic portal venous obstruction (EHPVO) is a common cause of variceal bleeding in India [1]. Mortality in these individuals results from uncontrolled variceal hemorrhage, as liver function is normal, as opposed to patients with cirrhosis of the liver. Though endoscopic variceal sclerotherapy has been successfully employed in achieving long term control of variceal bleeding in this condition [2, 3], certain patients require surgery due to failure of sclerotherapy, bleeding fundal gastric varices and hypersplenism. Spleno-renal shunt surgery has shown good results in patients with EHPVO [4,5]. However, 33-50% [1,6] of patients may have extensive thrombosis of the portal and splenic veins making them unsuitable for shunt surgery [6,7]. The management of these patients poses a challenge to the surgeon, some have even recommended drastic steps in the form of total esophagogastrectomy [7,8] in the management of this condition.

This study is an evaluation of extensive transabdominal esophago-gastric devascularisation with gastro-esophageal stapling (modified Sugiura's procedure) in the medium term

*Address for correspondence: 123, Khushnuma, 29A Carmichael Road, Bombay 400 026, India.

prevention of variceal bleeding in patients with EHPVO who have not received previous surgical therapy for portal hypertension and were unsuitable for orthodox spleno-renal shunt surgery.

### PATIENTS AND METHODS

Between January 1990 and May 1997, 14 patients of portal hypertension secondary to EHPVO where venous anatomy was unsuitable for orthodox shunt surgery were subjected to a modified Sugiura's devascularisation procedure. These patients had no previous operative intervention. All patients had a history of variceal hemorrhage. The surgery has performed as an emergency procedure in 5 patients – 3 were bleeding from fundal gastric varices and in two endoscopic variceal sclerotherapy had failed to control variceal hemorrhage. Nine patients were subjected to elective surgery, all for documented bleeding from diffuse fundal gastric varices not amenable to endoscopic management.

Detailed history was taken regarding previous bleeding episodes, treatment taken and possible etiology of EHPVO. Hemogram, liver function tests and upper GI endoscopy were performed in all cases.

Extensive thrombosis of the porto-splenic axis was conclusively demonstrated by evaluation of the portal system with the help of ultrasonography as well as percutaneous splenoportovenography in all cases.

The operative procedure was our modification of Sugiura's procedure [9]. The approach was through an upper midline incision. The upper two thirds of the stomach was devascularised, ligating the branches from the gastro-epiploic arcade, left gastro-epiploic, short gastric, retrogastric and left gastric veins. The lower 10 cm of the esophagus was devascularised by dividing the para-esophageal veins and the perforating veins to the esophageal wall. A stapled esophageal transection was done 2 cm above the esophago-gastric junction with an EEA stapler (Auto Suture Inc., Norwalk, USA) in 11 cases; in two cases, isolated gastric varices were present and in a three year old, the esophagus was too small for the smallest (No. 25) sizer, hence underruning of the esophageal varices was done instead. Fundal gastric varices were underrun with interlocking silk sutures. Splenectomy was not done in 13 cases; splenic artery ligation was performed in three patients with low platelet counts (< 100 000/cmm) and splenectomy was done in one patient who complained of dragging pain due to a large spleen. A floppy Nissen fundoplication was added in all cases. A liver biopsy was performed.

Follow up upper GI endoscopy was done at 6 monthly intervals to assess variceal status

Analysis was done for intra operative blood loss, operative time, post operative complications, variceal recurrence, rebleed and survival.

### RESULTS

Of the fourteen patients, there were 9 males and 5 females. The mean age at presentation was 17.2 years SD 12.8 years (range 3–48 years). The etiology of EHPVO was believed to be umbilical sepsis in three (21%). One patient had alcohol induced chronic pancreatitis, the pain of pancreatitis was controlled with enzymes. The patients had a mean of 2.5 SD 1.3 episodes of bleeding (range 1–5) prior to surgery and were transfused a mean of 1540 SD 950 ml of blood (range 350–3500 ml) prior to surgery. The mean spleen size was 5 cm SD 3.7 cm below the costal margin (range 0–12 cm). No patient had symptomatic hypersplenism in the form of bleeding gums, purpurae or recurrent respiratory tract infections.

Four of the five surgeries in the emergency setting were performed during the first hospital admission; one of these had failed therapy with cyanoacrylate glue injection for fundal varices before presentation to us. The fifth was for a patient with esophageal variceal rebleed after 14

sittings of endoscopic variceal sclerotherapy. Seven patients operated electively were not subjected to endoscopic variceal sclerotherapy as they had large fundal gastric varices on index endoscopy and two were patients who had esophageal varices obliterated and had secondarily developed fundal varices that had bled.

All patients had normal serum bilirubin levels and prothrombin times. The mean serum total protein levels were 6.0 SD 0.6 (5.3–7.2 g/dL) with serum albumin levels of 3.4 SD 0.3 (2.4–3.8 g/dL). The albumin: globulin ratio was > 1 in all patients. All intra-operative liver biopsies revealed normal liver architecture.

The median operative time was 240 min (150–480 min) and median blood loss was 500 ml (100–2200 ml). The blood loss was less than 1000 ml in all bar the patient with chronic pancreatitis where dense adhesions were present. One patient had an esophageal tear intraoperatively resulting from post sclerotherapy necrosis which was treated by excision of the affected segment of the esophagus.

Variceal bleeding ceased in all 5 patients operated in the emergency setting.

There was no 30 day mortality. Post operative complications included pneumonia in 2, subphrenic abscess in one, treated by percutaneous drainage, and esophageal leak in one patient treated conservatively. The mean post operative stay was 16 days SD 5.5 days (10–28 days).

Esophageal staple line strictures developed in two (14%) patients which responded to 1 and 2 sessions of endoscopic balloon dilatation.

On follow up endoscopy, at six months, 12 of thirteen patients (92%) completing this period were free of esophageal and gastric varices. One patient was operated three months ago. One patient had a residual subcardiac gastric varix as well as congestive gastropathy on follow up endoscopy which bled subsequently. An attempt to eradicate the varix with cyanoacrylate glue failed and the patient was subjected to a makeshift shunt between a large splenic collateral and the adrenal vein. The patient rebled 5 months later once again from the sub cardiac gastric varix. The shunt was patent but stenosed. Cyanoacrylate glue was re-injected into the varix and the patient was started on propranolol. After a month the repeat endoscopy showed disappearance of the varix and mild gastropathy. The remaining patients are free from varices. One patient was lost to follow up after the first check endoscopy, one bleed free and awaiting his first check endoscopy. The remaining 12 have followed up over a mean of 38.4 months SD 22.6 months (range 17–90 months). There has been no late mortality.

### DISCUSSION

Unshuntable portal hypertension is a challenge for the clinician. Recurrent admissions and massive blood transfusions are required in these patients if timely intervention is not undertaken [7]. Mortality may ensue from massive hemorrhage or from complications of blood transfusion in patients who would otherwise have a normal life expectancy as there is no liver dysfunction.

Endoscopic variceal sclerotherapy often does not provide an answer as patients bleed from large fundal varices. This has been reported in other series [7, 10] and in our series as well; 85% of our patients had diffuse fundal varices. Endoscopic variceal sclerotherapy has given poor results in the management of these varices. Cyanoacrylate glue, though effective in the immediate control of fundal variceal bleeding, may not prove to be a long term solution as recurrence is common [11] leaving surgery as the only answer.

Orthodox shunt surgery had little role to play. When the thrombus extends along the portosplenic axis, the chief route of decompression of the fundal gastric varices – the splenic vein, is blocked; splenorenal shunts are not possible and a shunt with a superior mesenteric vein or its branch, even if patent, will not achieve decompression of gastric varices. Unorthodox shunts though occasionally successful [12] have a high failure rate [13]. Even if the shunt remains patent, adequate decompression through the small collateral does not take place [12], as was seen in one patient in this series.

Non shunt surgeries, therefore, offer the best form of treatment. Limited devascularisation procedures have high rebleed rates [10] hence a more extensive procedure is required. The Sugiura's procedure has achieved great success in the treatment of EHPVO in Japan as well as outside [6]. It is the only form of treatment for unshuntable portal hypertension and has been used with good effect with a 9% rebleed rate [6]. The authors have earlier reported a modification of the Sugiura's procedure which provides comparable results of rebleed in patients with mixed etiology, with less operative time, blood loss and low post operative morbidity due to various modifications – the exclusively transabdominal approach, avoiding thoracotomy and preservation of the spleen without compromising on extensive venous disconnection and esophageal transection [9]. In the present study, this procedure has a low rebleed rate on follow up and has been shown to be highly effective in the management of this complex problem. A similar result has been reported in a smaller series of 8 patients [14]. Another series reported a 24% recurrence after an extensive devascularisation [10]. The higher recurrence in this series may be attributed to the failure to perform a stapled esophageal transection in these patients for fear of an esophageal leak. The intraoperative modifications to tackle the friable post sclerotherapy esophagus have been discussed by us earlier [9].

A more radical approach has been suggested by Orloff *et al.* [7] for achieving cure. He recommends total esophagogastrectomy with colonic bypass. This is a complex procedure requiring multiple anastomosis with a permanent alteration in gut physiology. The modified Sugiura's procedure described requires far less skill and operative time and can be carried out by any general surgeon with equivalent results. However, radical management may be required, as suggested by Orloff for patients subjected to multiple surgeries as adhesions may preclude a safe devascularisation or may result in an incomplete procedure and recurrence requiring further surgery. Series reporting patients with "unshuntable" portal hypertension include a majority who may previously have been shuntable but were subjected to surgery such as splenectomy [4,7]. The current series includes only those patients who present primarily with unshuntable disease.

In conclusion, in patients with primary "unshuntable" disease, a modified Sugiura's procedure of extensive esophagogastric devascularisation with stapled esophagogastric transection and underrunning of fundal gastric varices constitutes a swift, safe and satisfactory medium term solution.

### *References*

[1] Koshy, A., Bhasin, D. K. and Kapur, K. K. (1984). Bleeding in extra hepatic portal vein obstruction. *Indian Journal of Gastroenterology*, **3**, 13–14.

[2] Mathur, S. K., Naik, S. R., Supe, A. N., Plumber, S. T., Pipalia, D. and Bhalerao, R. A. (1992). Endoscopic variceal sclerotherapy using 3% aqueous phenol. *Gastrointestinal Endoscopy*, **38**, 152–157.

[3] Kahn, D., Krige, J. E., Terblanche, J., Bornman, P. C. and Robson, S. C. (1994). A 15-year experience of injection sclerotherapy in adult patients with extrahepatic portal venous obstruction. *Annals of Surgery*, **219**, 34–39.

[4] Warren, W. D., Henderson, J. M., Millikan, W. J., Galambos, J. T. and Bryan, F. C. (1988). Management of variceal bleeding in patients with non cirrhotic portal vein thrombosis. *Annals of Surgery*, **207**, 623–634.

[5] Prasad, A. S., Gupta, S., Kohli, V., Pande, G. K., Sahni, P. and Nundy, S. (1994). Proximal splenorenal shunts for extra hepatic portal venous obstruction in children. *Annals of Surgery*, **219**, 193–196.

[6] Orozco, H., Takahashi, T., Mercado, M., Prado, E. and Chan, C. (1994). Surgical management of extrahepatic portal hypertension and variceal bleeding. *World Journal of Surgery*, **18**, 246–250.

[7] Orloff, M. J., Orloff, M. S., Daily, P. O. and Girard, B. (1994). Long term results of radical esophagogastrectomy for bleeding varices due to unshuntable extra hepatic portal hypertension. *American Journal of Surgery*, **167**, 96–103.

[8] Cooley, D. A. and DeBakey, M. E. (1954). Subtotal esophagectomy for bleeding esophageal varices. *Archives of Surgery*, **68**, 854–871.

[9] Mathur, S. K., Shah, S. R., Soonawala, Z. F. *et al.* (1997). Transabdominal extensive oesophagogastric devascularisation with gastro-oesophageal stapling in the management of acute variceal bleeding. *British Journal of Surgery*, **84**, 413–417.
[10] Jin, G. and Rikkers, L. F. (1996). Transabdominal esophagogastric devascularisation as treatment for variceal hemorrhage. *Surgery*, **120**, 641–647.
[11] Oho, K., Iwao, T., Sumino, M., Toyonaga, A. and Tanikawa, K. (1995). Ethanolamine oleate *versus* butyl cyanoacrylate for bleeding gastric varices: a non randomised study. *Endoscopy*, **27**, 349–354.
[12] D'Cruz, A., Kamath, P. S., Ramchandra, C. and Jalihal, A. (1995). Non-conventional portosystemic shunts in children with extra hepatic portal vein obstruction. *Acta Paediatrica Japonica*, **37**, 17–20.
[13] Fonkalsrud, E. W., Myers, N. A. and Robinson, M. J. (1974). Management of extrahepatic portal hypertension in children. *Annals of Surgery*, **180**, 487–493.
[14] Caps, M. T., Helton, W. S. and Johansen, K. (1996). Left-upper-quadrant devascularization for 'unshuntable' portal hypertension. *Archives of Surgery*, **131**, 834–838.

### COMMENTARY

Portal hypertension due to extrahepatic portal venous obstruction is rarely observed in Europe. In contrast such pathology is frequently observed in developing countries. As outlined by Shah *et al.*, the potential therapies for variceal bleeding due to this pathology are scarce and remain relatively disappointing. The technique previously described by Shah *et al.* (a modified Sugiura's devascularisation procedure) seems to offer a good response to this situation. However, it must be outlined that this study is an open one including a limited number of patients. Moreover, one failure was observed and a long-term follow-up is still lacking. This last point must be provided in the future since this pathology and its complications are observed in young patients.

Finally, as outlined by the authors, their surgical technique should be restricted to patients with portal hypertension due to extrahepatic portal venous obstruction and unsuitable for orthodox spleno-renal shunt surgery. The absence of previous abdominal surgery seems also very important. In other situation (*i.e.*, possibility to perform a spleno-renal shunt), the modified Sugiura procedure can not be recommended before obtaining the results of a controlled trial.

Prof. Y. Horsmans
Universite Catholique de Louvain
Cliniques Universitaire Saint-Luc
Avenue Hippocrate 10
1200 Bruxelles
Belgium

